# *Mycobacterium tuberculosis* infects human peripheral hematopoietic multipotent progenitors and promotes their myeloid differentiation in vitro

**DOI:** 10.1038/s41419-026-09122-0

**Published:** 2026-07-31

**Authors:** Tonino Alonzi, Daniela Peruzzu, Alessandra Aiello, Giulio Bontempi, Chiara Farroni, Federica Repele, Settimia Sbarra, Valentina Vanini, Ivana Palucci, Michela Sali, Luca Pierelli, Raffaele Strippoli, Giovanni Delogu, Delia Goletti

**Affiliations:** 1 Translational Research Unit, National Institute for Infectious Diseases “Lazzaro Spallanzani”-IRCCS, Rome, Italy; 2 Department of Epidemiology, Preclinical Research and Advanced Diagnostics, National Institute for Infectious Diseases IRCCS “Lazzaro Spallanzani”, Rome, Italy; 3 Department of Molecular Medicine, University of Rome “Sapienza”, Rome, Italy; 4 Unità Operativa Semplice (UOS) Professioni Sanitarie Tecniche, National Institute for Infectious Diseases Lazzaro Spallanzani-IRCCS, Rome, Italy; 5 Dipartimento di Scienze Biotecnologiche di base, Cliniche Intensivologiche e Perioperatorie, Università Cattolica del Sacro Cuore, Rome, Italy; 6 Department of Laboratory Sciences and Infectious Diseases, Fondazione Policlinico Universitario “A. Gemelli”, 00168 Rome, Italy; 7 Transfusion Medicine Unit, San Camillo Forlanini Hospital, Rome, Italy; 8 Department of Experimental Medicine, Sapienza University, Rome, Italy; 9 Department of Medicine and Surgery, University of Parma, Parma, Italy; 10 Unit of Microbiology and Virology, University Hospital AO Parma, Parma, Italy

**Keywords:** Infection, Tuberculosis

## Abstract

*Mycobacterium tuberculosis* (*Mtb*) persists as a global health threat partly by establishing long-term reservoirs in myeloid cells. Although progenitors have been proposed as additional sites of persistence and immune reprogramming, it remains unclear whether circulating human hematopoietic stem/progenitor cells (HSPC) are permissive to *Mtb* infection. Here, we show that peripheral blood-derived CD34^+^ HSPC are permissive to *Mtb*, in vitro. Confocal microscopy showed *Mtb* cytoplasmic localization, while colony-forming unit (CFU) quantification demonstrated early intracellular *Mtb* growth. Flow cytometry revealed preferential *Mtb* infection of multipotent progenitors (MPP) with progressive enrichment of granulocyte-monocyte progenitors (GMP) and lineage-committed cells, indicating myeloid skewing. Infection drove early IL-8 and TNF-α release, followed by MIP-1α, MIP-1β, and IP-10, demonstrating a dynamic inflammatory response by CD34^+^ progenitors. Parallel infection experiments with *Mycobacterium bovis* BCG revealed that, despite lacking key virulence determinants, BCG infects CD34^+^ cells in vitro with a preference for MPP. Unlike *Mtb*, BCG infection did not promote expansion of more differentiated progenitor subsets, indicating that virulence-dependent mechanisms are required to drive progenitor differentiation and myeloid skewing. These findings identify CD34^+^ HSPC as a permissive niche for mycobacterial infection and suggest that *Mtb*-specific virulence factors contribute to the modulation of progenitor differentiation. Further studies are needed to fully assess the consequences of these events on the systemic immune modulation observed during tuberculosis infection.

## Introduction

*Mycobacterium tuberculosis* (*Mtb*), the causative agent of tuberculosis (TB), remains one of the leading causes of death from a single infectious agent worldwide [1]. Despite the availability of effective preventive therapy, TB control is hindered by the capacity of *Mtb* to establish long-term persistence within the host, resulting in a chronic infection previously called “latent infection”, which can reactivate [2, 3]. This persistence relies largely on the ability of *Mtb* to infect host cells and subvert antimicrobial mechanisms, thereby evading immune clearance and reshaping host immune responses [4–6].

Macrophages are the primary cellular targets of *Mtb* following inhalation of aerosolized bacilli into the lung [7–10]. Alveolar macrophages internalize *Mtb* but frequently fail to eliminate it, as the pathogen actively inhibits phagosome maturation and resists intracellular killing pathways [2, 3]. Infected macrophages provide a niche for bacterial persistence and are central to granuloma formation, the pathological hallmark of TB [4, 5]. In addition to macrophages, other myeloid cells, including monocytes, dendritic cells, and neutrophils, can be infected and contribute to bacterial dissemination, antigen presentation, and immunopathology [4, 11, 12].

Beyond professional phagocytes, several studies have demonstrated that non-myeloid cells are also susceptible to *Mtb* infection. Alveolar epithelial cells can internalize *Mtb* and support intracellular survival, suggesting a role in early infection events and local immune modulation [13–16]. More recently, increasing attention has focused on the bone marrow as a potential reservoir for *Mtb*. Experimental and clinical evidence indicate that *Mtb* can infect hematopoietic stem and progenitor cells (HSPC), including CD34^+^ cells [17–21].

Infection of HSPC has been proposed as a mechanism for long-term bacterial persistence and immune system reprogramming, linking TB with chronic inflammation and sustained immune dysregulation [22–24]. Notably, *Mtb* DNA has been detected in circulating CD34^+^ peripheral blood mononuclear cells of individuals with TB infection, disease, and in the bone marrow of cured TB disease patients, providing direct clinical evidence that progenitor cells are involved in human TB pathogenesis [17, 19, 21]. A comprehensive understanding of the cellular niches that support *Mtb* persistence is therefore essential for elucidating the mechanisms underlying latency, reactivation, and disease progression.

Previous research has shown that *Mtb* can infect CD34^+^ cells derived from umbilical cord blood in vitro [18, 25]. Nevertheless, whether *Mtb* can infect human peripheral HSPC, and whether such infection promotes differentiation within HSPC or selectively targets specific cell populations, remains to be elucidated. In this study, we investigated the capacity of *Mtb* to infect human peripheral CD34^+^ HSPC in vitro and sought to characterize the phenotype of these *Mtb*-infected cells.

## Results

### *Mtb* infects circulating human CD34^+^ HSPC in vitro

**Fig. 1:**
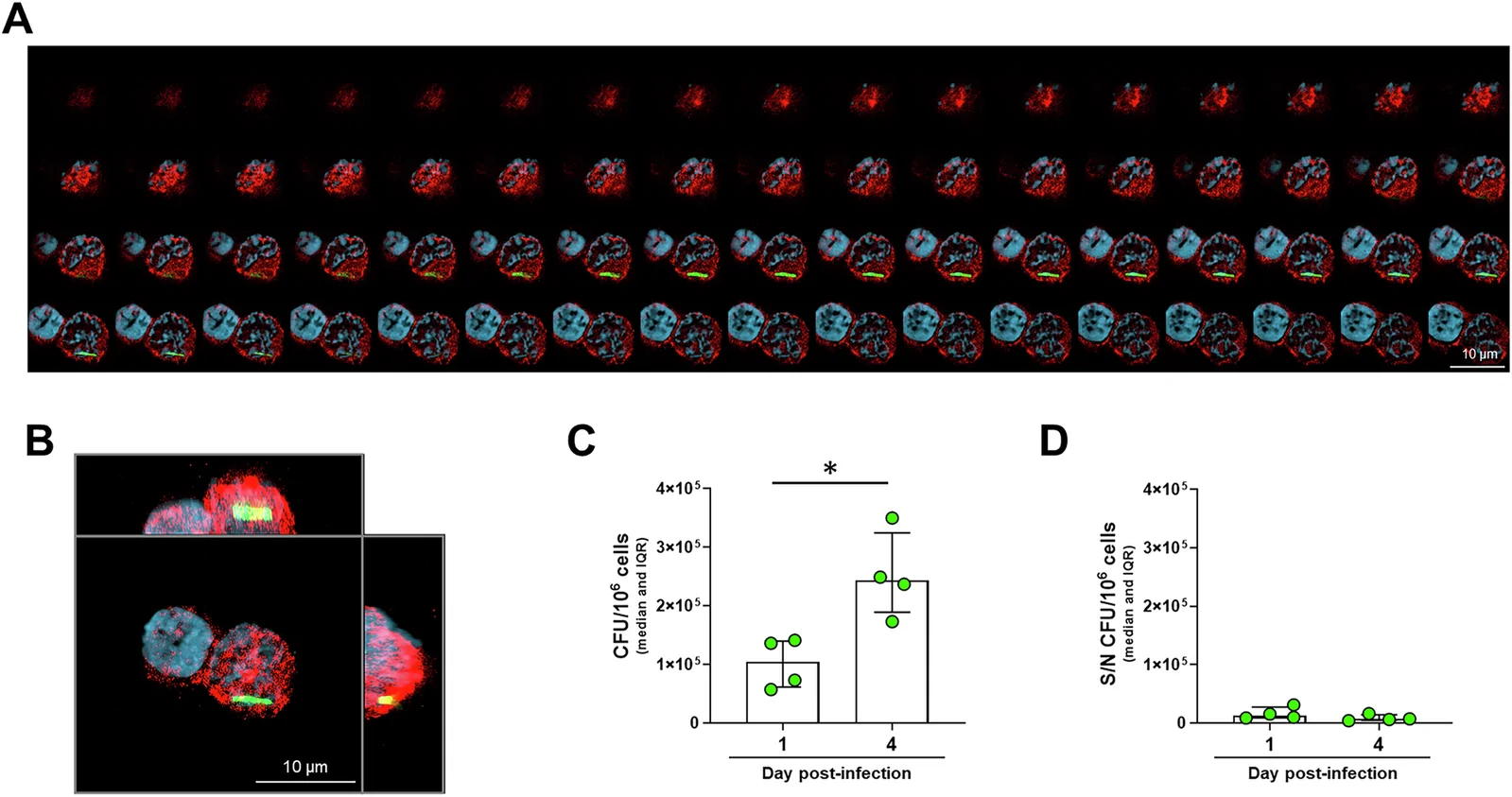
Intracellular localization of *Mtb*-GFP in circulating human CD34^+^ HSPC. **A, B** Confocal z-stack images of CD34^+^ cells labeled with an anti-CD34 (red signal) and for nuclei (blue signal), showing the presence of *Mtb*-GFP bacilli (green signal). **A** Sequential optical sections demonstrate that the GFP signal is fully enclosed between the plasma membrane (CD34, red signal) and nucleus (blue signal), indicating intracellular localization. **B** Orthogonal x–z and y–z views confirming that *Mtb*-GFP resides within the cytoplasmic compartment. **C** Intracellular and **D** extracellular colony‑forming unit (CFU) at days 1 and 4 post-infection. CFU/10^6^ cells are reported as median with interquartile range (IQR) from four independent experiments (CD34^+^ cells were isolated from four independent pooled buffy coats, each derived from five donors). Statistical analysis was performed using GraphPad Prism Software v.8, and data were analysed using the unpaired nonparametric Mann-Whitney U test. Statistical significance was defined as: (*) *p* < 0.05. S/N: Supernatant.

To assess whether *Mtb* infects human peripheral CD34^+^ HSPC, we isolated these cells from buffy coats obtained from healthy donors. CD34^+^ HSPC were enriched from peripheral blood mononuclear cells (PBMC) by immunomagnetic positive selection, achieving 95–98% purity (Supplementary Fig. 1), and subsequently infected in vitro with *Mtb*, as schematically shown in supplementary Fig. 2. To enable direct visualization of *Mtb* during infection, we used a genetically modified derivative of the H37Rv laboratory reference strain engineered to constitutively express the green fluorescent protein (*Mtb*-GFP). CD34^+^ HSPC were infected with *Mtb*-GFP at a multiplicity of infection (MOI) of 5:1 (MOI = 5) for 6 h (h), after which confocal microscopy was performed. Before imaging, cells were stained with an antibody against CD34, which is preferentially located at the plasma membrane, and with a nuclear dye to visualize nuclei. High‑resolution z‑stack confocal images showed a GFP staining positioned between the surface CD34 red signal and the blue nuclear stain, thus revealing an intracellular localization of *Mtb*-GFP unambiguously, while excluding a mere adherence to the plasma membrane in human CD34⁺ cells (Fig. 1A). Orthogonal (x–z and y–z) views confirmed that the *Mtb*-GFP bacilli are located within the cytoplasmic compartment, clearly separated from the plasma membrane and situated around the nucleus (Fig. 1B). A 360° rotational 3D reconstruction video of the z-stack further illustrates this intracellular localization, showing the CD34⁺ cells rotating on all axes and demonstrating that the *Mtb*-GFP signal stays entirely enclosed by the plasma membrane and remains outside the nuclear area during the full rotation (Supplementary Video 1).

Because anti-CD34 staining can label not only the plasma membrane but also intracellular CD34 that has not yet been trafficked or degraded, potentially confounding localization, we used a membrane-specific stain (WGA Alexa Fluor 488) to better define the cell surface. Because this signal overlaps with *Mtb*-GFP fluorescence, we used an *Mtb* DsRed, that stably express the red fluorescent protein mCherry, thus enabling us to unequivocally visualize *Mtb* as intracellular (Supplementary Fig. 3).

These results were also confirmed in cells stained with a membrane-specific dye infected with a *Mtb* DsRed as a marker (Supplementary Fig. 3A). Collectively, these analyses show that *Mtb* infects CD34^+^ HSPC in vitro.

To investigate whether *Mtb* survives within human CD34^+^ cells, we performed a colony-forming unit (CFU) assay. To ensure that these results were not biased by residual extracellular bacteria, CD34^+^ cells were infected with *Mtb*‑GFP for 6 h and treated with gentamicin (50 μg/mL for 2 h), a condition commonly used to eliminate extracellular mycobacteria while preserving HSPC integrity. CFU counts of recoverable *Mtb*-GFP significantly increased by an average of about 0.5 log_10_ over time post-infection (Fig. 1C). Moreover, viable bacilli were measured in culture supernatants at day 1 and day 4 post-infection. CFU analysis revealed a marked reduction in extracellular bacteria, with counts remaining more than 1 log lower than intracellular CFU levels at day 1 and ~30-fold lower at day 4 post-infection (Fig. 1D).

Collectively, these results demonstrate the presence of live intracellular bacilli within CD34^+^ cells during the early stages of infection (until 4 days post-infection), suggesting that CD34^+^ hematopoietic progenitors may serve as a permissive niche supporting *Mtb* survival and replication.

### *Mtb* primarily infects peripheral human CD34^+^ HSPC in vitro at the differentiation stage of multipotent progenitors (MPP)

Next, we aimed to quantify the frequency of *Mtb*‑infected CD34^+^ cells and identify the specific differentiation stage at which CD34^+^ cells are more susceptible to *Mtb* infection. We accomplished this by flow cytometry (the gating strategy is shown in Supplementary Fig. 4). CD34^+^ cells were in vitro infected with *Mtb*-GFP (MOI = 5) for 6 h, gentamicin treated (50 µg/mL, 2 h), washed, and cultured for 1 and 4 days.

**Fig. 2:**
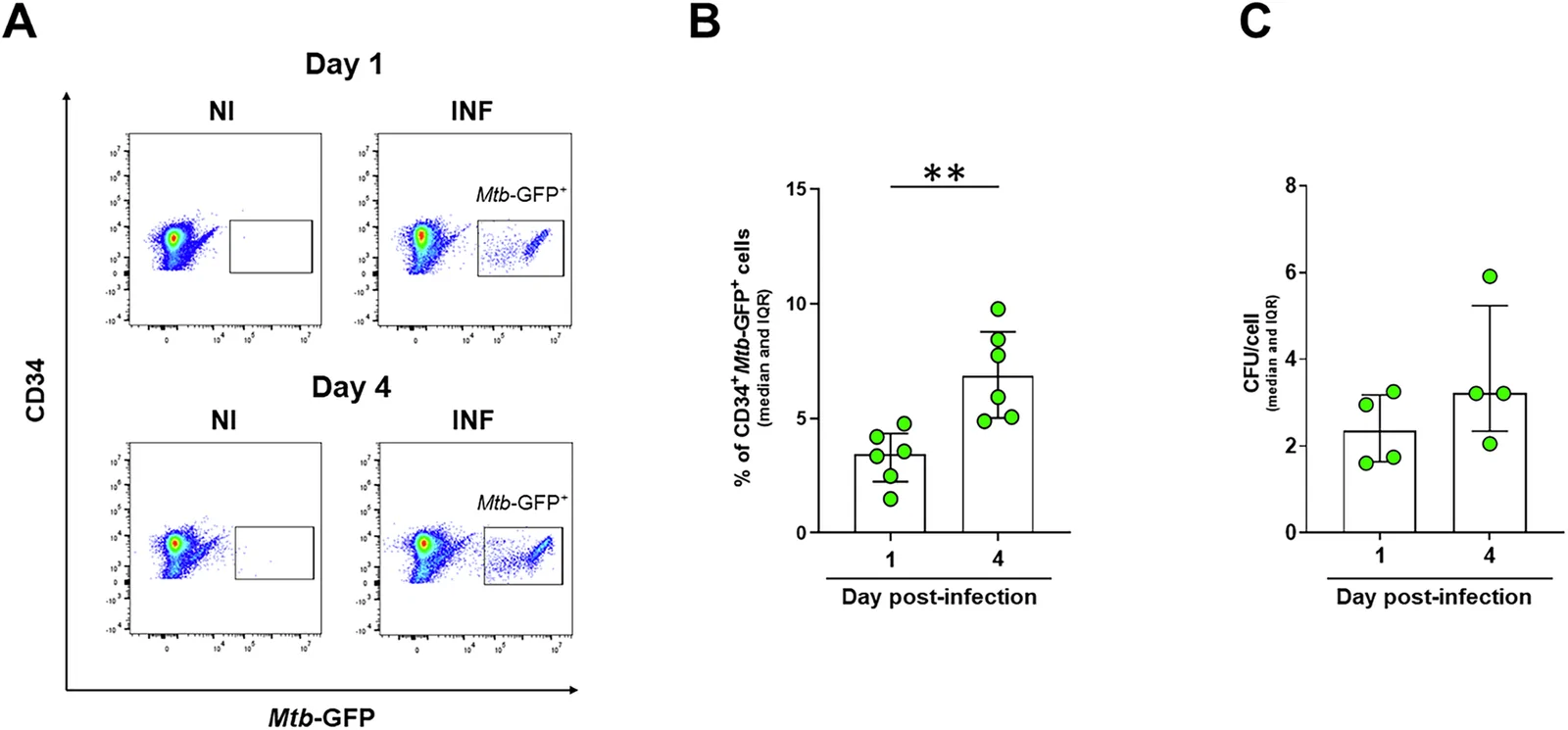
*Mtb*-GFP infects CD34^+^ cells in vitro. **A** Representative flow cytometry plots showing *Mtb*-GFP^+^ cells within the CD34^+^ population in uninfected (NI) and *Mtb*-infected (INF) cultures at day 1 and 4 post-infection. **B** Frequency of CD34^+^ *Mtb*-GFP^+^ cells in INF cultures at day 1 and day 4 post-infection. Data are expressed as median with interquartile range (IQR) from six independent experiments (CD34^+^ cells were isolated from six independent pooled buffy coats, each derived from five donors). **C** Colony‑forming unit (CFU) assay at days 1 and 4 post-infection. CFU/cells values are reported as median with IQR from four independent experiments (CD34^+^ cells were isolated from four independent pooled buffy coats, each derived from five donors). Statistical analysis was performed using GraphPad Prism Software v.8, and data were analysed using the nonparametric Mann-Whitney U test. Statistical significance was defined as (**) *p* < 0.01.

Since washing and gentamicin treatment were used to remove extracellular bacteria, and exclude the presence of bacilli attached to the cell surface, the frequency of live CD34^+^ *Mtb*-GFP^+^ cells was taken as a measure of the *Mtb* infection rate (Fig. 2A). At day-1 post-infection, 3.5% (interquartile range [IQR]: 2.2-4.3) of viable CD34^+^ cells were *Mtb*-GFP^+^, and at day 4 post-infection, this proportion increased to 6.8% (IQR: 5.0–8.7; *p* = 0.002) (Fig. 2B; Supplementary Fig. 5). Cell viability analyses showed that the proportion of dead cells increased comparably over time in both uninfected and infected CD34^+^ cells (Supplementary Fig. 6), suggesting that *Mtb* infection did not substantially affect overall viability.

Since confocal microscopy experiments demonstrated that cells could contain multiple bacilli (Supplementary Fig. 3), we estimated the average number of *Mtb*‑GFP bacilli per CD34^+^ cell by measuring CFU and the number of living CD34^+^ cells. As shown in Fig. 2C, the median number of *Mtb*-GFP per CD34^+^ cell increased from 2.35 at day 1 post‑infection to 3.21 at day 4 post‑infection, thereby suggesting a possible intracellular *Mtb* replication.

By flow cytometry, we identified the HSPC differentiation stages most permissive to *Mtb* infection using an immunophenotyping panel designed to identify human hematopoietic progenitor and lineage‑committed populations. Progenitor subsets were defined according to established marker combinations (Table 1) [26, 27].

**Table 1:** Immunophenotypic profiling of CD34^+^ hematopoietic stem and progenitor cells.

CD34^+^ Hematopoietic Stem and Progenitor cells (HSPC)	Phenotype
**Hematopoietic stem cells (HSC)**	Lin-; CD34^+^; CD38^−^; CD45RA^−^; CD90^+^
**Multipotent (MPP)**	Lin^−^; CD34^+^; CD38^−^; CD45RA^−^; CD90^−^
**Multipotent Lymphoid (MLP)**	Lin^−^; CD34^+^; CD38^−^; CD45RA^+^; CD90^−^; CD10^+^
**Common Myeloid (CMP)**	Lin^−^; CD34^+^; CD38^+^; CD45RA^−^; CD10^−^
**Common Lymphoid (CLP)**	Lin^−^; CD34^+^; CD38^+^; CD45RA^+^; CD10^+^
**Megakaryocyte/ Erythroid (MEP)**	Lin^−^; CD34^+^; CD38^+^; CD45RA^−^; CD10^−^; CD110^+^
**Granulocytes/ Macrophage (GMP)**	Lin^−^; CD34^+^; CD38^+^; CD45RA^+^; CD10^−^
**Committed Monocytic**	CD34^+^; CD45RA^+^; CD14^+^
**Committed Lineages**	CD34^+^; CD45RA^+^; CD3^+^ or CD19^+^ or CD56^+^ or CD16^+^

**Fig. 3:**
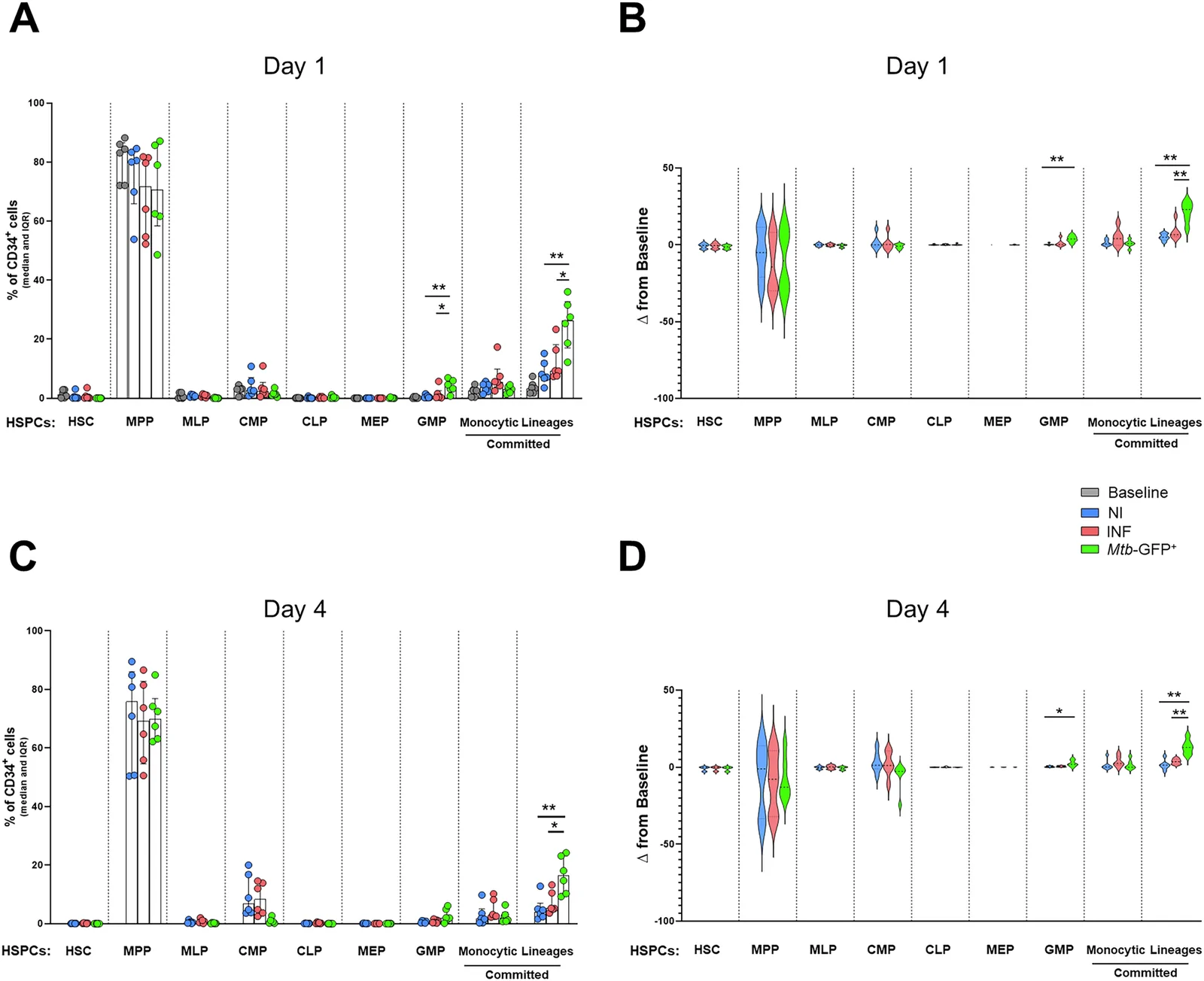
*Mtb* infected CD34^+^ cells with a multipotent progenitor (MPP) and lineage-committed differentiation stages. **A, C** Subset-specific analysis of uninfected (NI; blue), *Mtb*-infected (INF; red) and *Mtb*‑GFP^+^ CD34^+^ (green) cells at day 1 **A** and day 4 **C** post-infection compared to the day of CD34^+^ isolation (baseline; grey). Frequencies of each HSPC subset are shown as median with interquartile range (IQR) from six independent experiments (CD34^+^ cells were isolated from six independent pooled buffy coats, each derived from five donors). **B,** **D** Violin plots showing the changes (Δ) from baseline for NI (blue), INF (red) and *Mtb*‑GFP^+^ (green) CD34^+^ cells, calculated as the difference between the percentage measured at day 1 **B** or day 4 **D** post-infection and the corresponding baseline value. Statistical analysis was performed using GraphPad Prism Software v.8, and data were analysed using the nonparametric Mann-Whitney U test. Statistical significance was defined as: (*) *p* < 0.05, (**) *p* < 0.01. HSPC Hematopoietic Stem and Progenitor cells, HSC Hematopoietic Stem Cells, MPP Multipotent Progenitors, MLP Multipotent Lymphoid Progenitors, CMP Common Myeloid Progenitors, CLP Common Lymphoid Progenitors, MEP Megakaryocyte/ Erythroid Progenitors, GMP Granulocytes/ Macrophage Progenitors.

At baseline, CD34^+^ cells were predominantly composed of multipotent progenitors (MPP) (83.7%, IQR: 72.1–86.5), whereas HSC, downstream myeloid and lymphoid progenitors, monocytic progenitors, and lineage‑committed cells represented only minor fractions (Fig. 3A; grey dots). After 1 day of culture, a comparison was made between progenitor subset frequencies at baseline, in uninfected (NI), and in infected cells (INF). The analysis of CD34^+^ cells indicated that primitive progenitors [HSC, MPP, multilymphoid progenitors (MLP)] remained mostly stable across all conditions, with MPP being the predominant subset. No significant differences were observed between NI and INF cultures in all other differentiation stages (Fig. 3A; blue and red dots, respectively). Common myeloid progenitors (CMP), megakaryocyte–erythroid progenitors (MEP), (common lymphoid progenitors CLP), and monocytic committed subset showed minimal temporal variation across culture conditions (Fig. 3A and B).

Notably, the majority of CD34^+^ cells infected by *Mtb* (*Mtb*-GFP^+^) were found primarily within the MPP subset (MPP-*Mtb*-GFP^+^), followed by lineage-committed cells and, to a lesser extent, granulocyte-monocyte progenitors (GMP) (Fig. 3A; green dots). It is important to note that the percentage of GMP-*Mtb*-GFP^+^ cells and lineage-committed-*Mtb*-GFP^+^ was significantly increased with respect to those of NI and INF CD34^+^ populations (Fig. 3A and B; green dots and green violin plots, respectively). These findings suggest that *Mtb* infection accelerates the differentiation of CD34^+^ MPP, likely promoting their skewing toward the myeloid lineage. This hypothesis is supported by flow cytometry analyses conducted after four days of culture, where GMP-*Mtb*-GFP^+^ cells and lineage-committed *Mtb*-GFP^+^ cells remained significantly higher compared to NI and INF CD34^+^ populations (Fig. 3C and D).

**Fig. 4:**
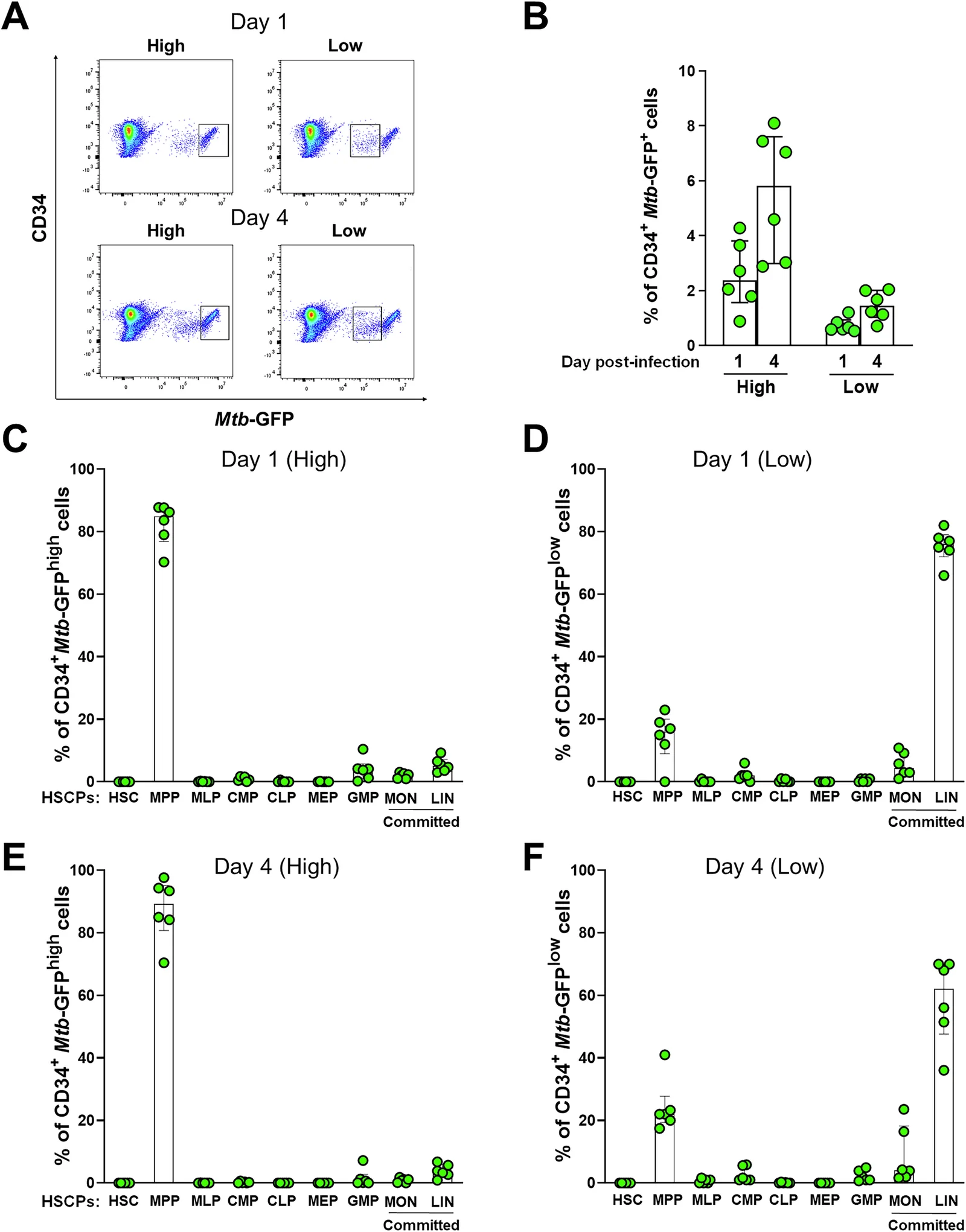
Multipotent progenitors (MPP) exhibit a GFP^high^ phenotype, whereas lineage‑committed CD34^+^ cells are GFP^low^. **A** Representative flow cytometry plots showing *Mtb*-GFP^high^ and *Mtb*‑GFP^low^ cells within the CD34^+^ population at day 1 and day 4 post-infection. **B** Frequency of *Mtb*-GFP^high^ and *Mtb*‑GFP^low^ CD34^+^ cells at day 1 and day 4 post-infection. **C–F** Subset-specific analysis of *Mtb*‑GFP^high^ **C, E** and *Mtb*‑GFP^low^ **D, F** CD34^+^ cells at day 1 **C, D** and day 4 **E, F** post-infection. Data are reported as median with interquartile range (IQR) from six independent experiments (CD34^+^ cells were isolated from six independent pooled buffy coats, each derived from five donors). HSPC Hematopoietic Stem and Progenitor cells, HSC Hematopoietic Stem Cells, MPP Multipotent Progenitors, MLP Multipotent Lymphoid Progenitors, CMP Common Myeloid Progenitors, CLP Common Lymphoid Progenitors, MEP Megakaryocyte/ Erythroid Progenitors, GMP Granulocytes/ Macrophage Progenitors, MON Monocytic, LIN Lineage.

Flow cytometry analysis showed that the CD34^+^ *Mtb*-GFP^+^ cells have two distinct levels of GFP intensity, which we defined as CD34^+^ *Mtb-*GFP^high^ and CD34^+^ *Mtb-*GFP^low^ populations (Fig. 4A). Variations in GFP intensity among CD34^+^ cells are likely directly proportional to the number of bacilli within each cell and may reflect differences in susceptibility to *Mtb* infection and/or the intracellular amount of *Mtb*. The *Mtb-*GFP^high^ population was the most abundant, with a median frequency of 2.4% (IQR: 1.6–3.8) at day 1 post-infection, increasing to 5.8% (IQR: 2.9–7.6) by day 4 (Fig. 4B). Different from the *Mtb-*GFP^high^ population, the frequency of the CD34^+^ *Mtb-*GFP^low^ population was low at day 1 post-infection (median frequency of 0.6%, IQR: 0.5–0.9), increasing modestly (1.4%, IQR: 1.0–2.0) at day 4 post-infection (Fig. 4B).

To better characterize these two *Mtb-*GFP populations, we analysed the co-expression of the different surface markers using the gating strategy shown in the supplementary Fig. 4. Analysis of this subset revealed that the majority of *Mtb-*GFP^high^ cells were classified as MPP at both day 1 (85%, IQR: 77–87) and day 4 (89%, IQR: 81–95) post-infection (Fig. 4C and E).

In contrast, most *Mtb-*GFP^low^ cells on day 1 were mainly lineage-committed CD34^+^ cells (76%, IQR: 72–79), followed by MPP (16%, IQR: 9–20) and monocytic progenitors (4.4%, IQR: 2.5–9.5). By day 4 post-infection, the levels of lineage-committed cells (62%, IQR: 48–70), MPP (22%, IQR: 19–28) and monocytic progenitors (4.0%, IQR: 1.8–18) changed little. These findings further support the idea that *Mtb* infection promotes differentiation of CD34^+^ progenitors toward the myeloid lineage (Fig. 4D and F).

### *Mtb*-infection induces the production of several immune factors in CD34^+^ cells

**Fig. 5:**
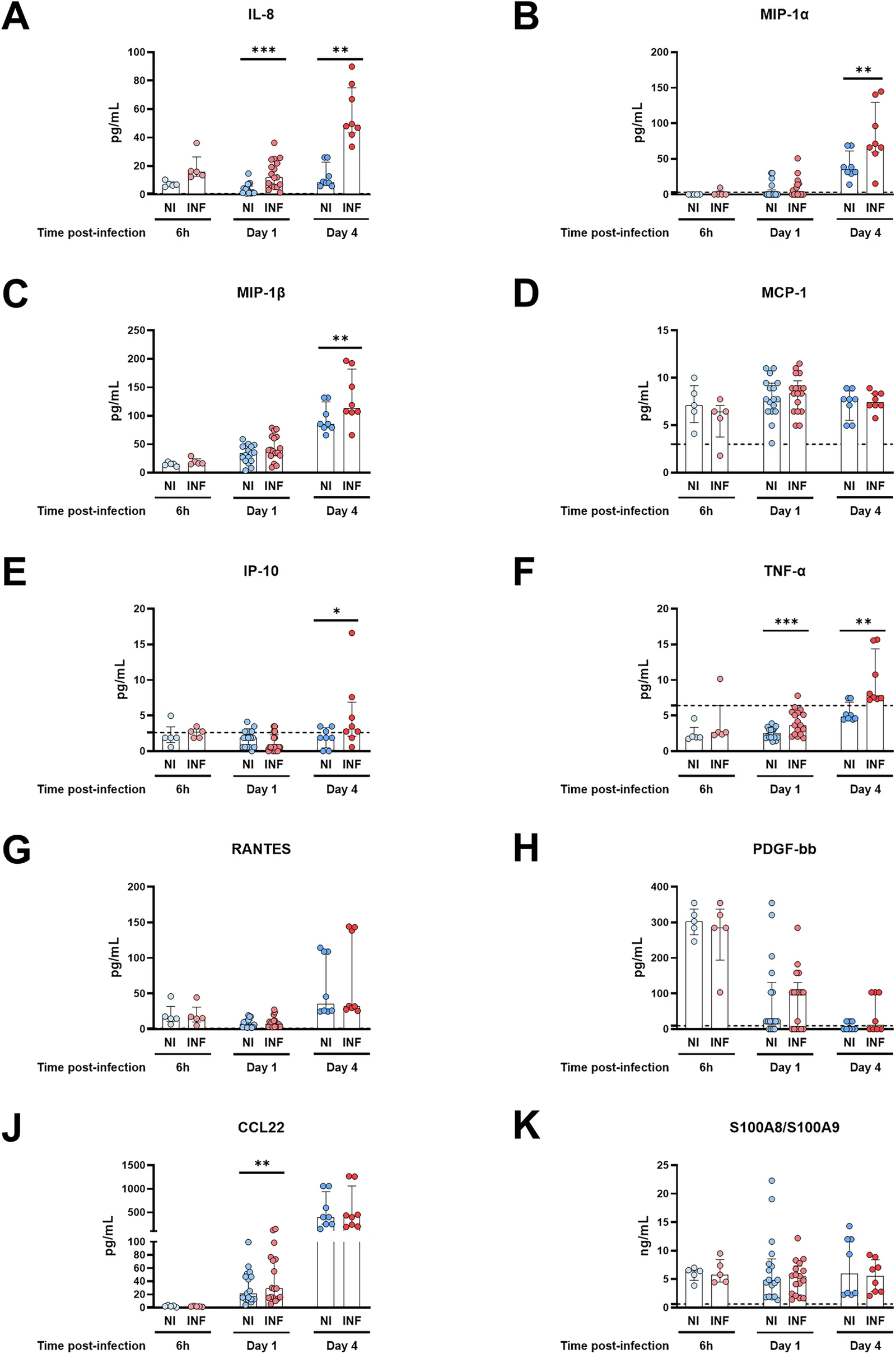
Immune factors released by CD34^+^ cells in response to *Mtb* infection. CD34^+^ cells were infected with *Mtb* (MOI = 5) for 6 h (h). Supernatants were collected at 6 h, day 1 and day 4 post-infection, and analysed for cytokine/chemokine production. **A–J** Values are expressed in pg/mL, except for S100A8/S100A9 **K**, which is reported in ng/mL. Black dashed lines indicate the lower limit of detection for each factor (IL-8: 0.64 pg/ml, MIP-1α: 3 pg/mL, MIP-1β: 0.38 pg/mL, MCP-1: 3 pg/mL, IP-10: 2.6 pg/mL, TNF-α: 6.4 pg/mL, RANTES: 1.3 pg/mL, PDGF-bb: 9.6 pg/mL, CCL22: 0.64 pg/mL, and S100A8/S100A9: 0.625 ng/mL). Data are presented as scatter plots with bars indicating the median and interquartile range (IQR) from five (6 h), seventeen (day 1) and eight (day 4) independent experiments (CD34^+^ cells were isolated from seventeen independent pooled buffy coats, each derived from five donors). Blue-scale dots represent uninfected CD34^+^ cells, whereas red-scale dots indicate *Mtb*-infected CD34^+^ cells. Statistical analysis was performed using the Wilcoxon signed-rank test, and significance was defined as: (*) *p* < 0.05, (**) *p* < 0.01, (***) *p* < 0.001. IL Interleukin, INF infected; IP-10: Interferon-γ-induced protein 10, MCP monocyte chemoattractant protein, MIP macrophage inflammatory protein, NI uninfected, PDGF platelet-derived growth factor, RANTES regulated on activation, normal T cell expressed and secreted, TNF tumor necrosis factor.

To identify the broadest spectrum of immune factors released by CD34^+^ cells in response to *Mtb*- infection, culture supernatants collected at 6 h, 1 day, and 4 days post-infection were analysed using multiple assays capable of detecting up to 38 analytes. We reported the results for the 10 analytes whose concentrations were above the lower limit of detection, indicative of effective production by the cells. Among the immune factors analysed, NI CD34^+^ cells constitutively produced interleukin (IL)-8, macrophage inflammatory protein (MIP)-1α, MIP-1β, monocyte chemoattractant protein-1 (MCP-1), regulated on activation, normal T cell expressed and secreted (RANTES), platelet-derived growth factor (PDGF)-bb, CCL22 and S100A8/S100A9 heterodimer that were either increased (IL-8, MIP-1α, MIP-1β, RANTES, CCL22) or unchanged (MCP-1, PDGF-bb, S100A8/S100A9) during the culture time (Fig. 5).

Conversely, when compared to NI cells, INF CD34^+^ cells significantly increased IL-8 (*p* < 0.0001) and induced Tumor Necrosis Factor-alpha (TNF-α) (*p* < 0.0001) levels as early as day 1 post-infection, and this response persisted at day 4 post-infection (both *p* = 0.008). In contrast, chemokines such as MIP-1α (*p* = 0.008), MIP-1β (*p* = 0.008), and Interferon gamma-induced protein (IP)-10 (*p* = 0.031) were significantly increased only at day 4 post-infection. No significant differences were found in the levels of MCP-1, RANTES, PDGF-bb, or S100A8/S100A9.

**Fig. 6:**
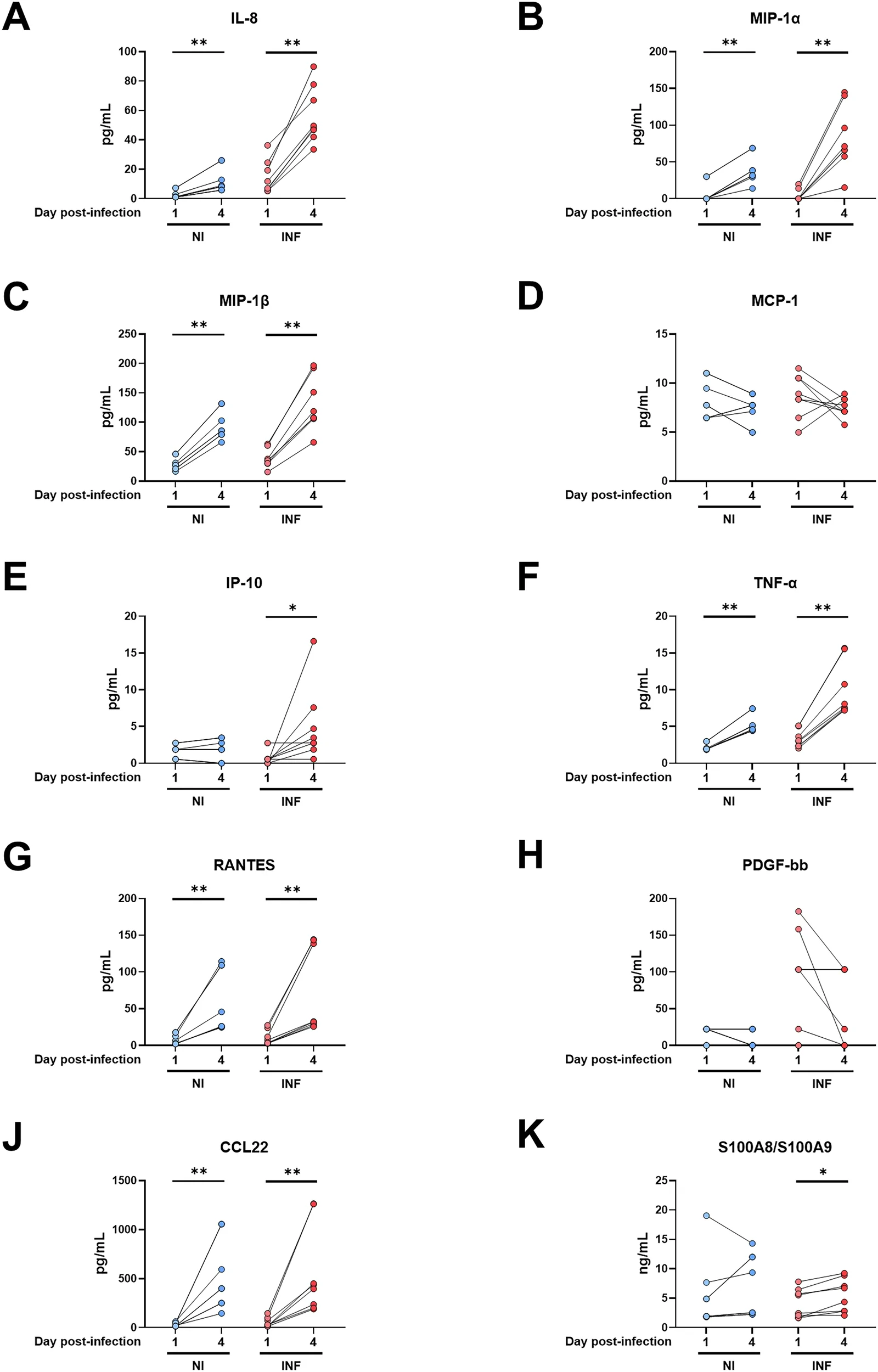
Time‑dependent production of immune factors by CD34^+^ cells induced by *Mtb* infection. Supernatants harvested from CD34^+^ cells infected with *Mtb* (MOI = 5) for 6 h were collected at day 1 and day 4 post-infection, and analysed for cytokine/chemokine production. **A–K** Values are expressed in pg/mL, except for S100A8/S100A9, which is reported in ng/mL. Data are presented as paired line charts from eight independent experiments (CD34^+^ cells were isolated from eight independent pooled buffy coats, each derived from five donors). Blue-scale dots represent uninfected CD34^+^ cells, whereas red-scale dots indicate *Mtb*-infected CD34^+^ cells. Statistical analysis was performed using the Wilcoxon signed-rank test, and significance was defined as: (*) *p* < 0.05, (**) *p* < 0.01. IL Interleukin, INF infected, IP-10 Interferon-gamma-induced protein 10, MCP monocyte chemoattractant protein, MIP macrophage inflammatory protein, NI uninfected, PDGF platelet-derived growth factor, RANTES regulated on activation, normal T cell expressed and secreted, TNF tumor necrosis factor.

In both uninfected and infected cells, longitudinal analysis of immune factor levels at days 1 and 4 post-infection revealed a significant longitudinal increased IL-8, MIP-1α, MIP-1β, TNF-α, RANTES, and CCL22 (all *p* = 0.008) production. In contrast, IP-10 (*p* = 0.031) and S100A8/S100A9 (*p* = 0.016) exhibited significant increases over time exclusively in infected cells (Fig. 6).

### BCG primarily infects in vitro CD34^+^ multipotent progenitors (MPP) without inducing their differentiation stage

**Fig. 7:**
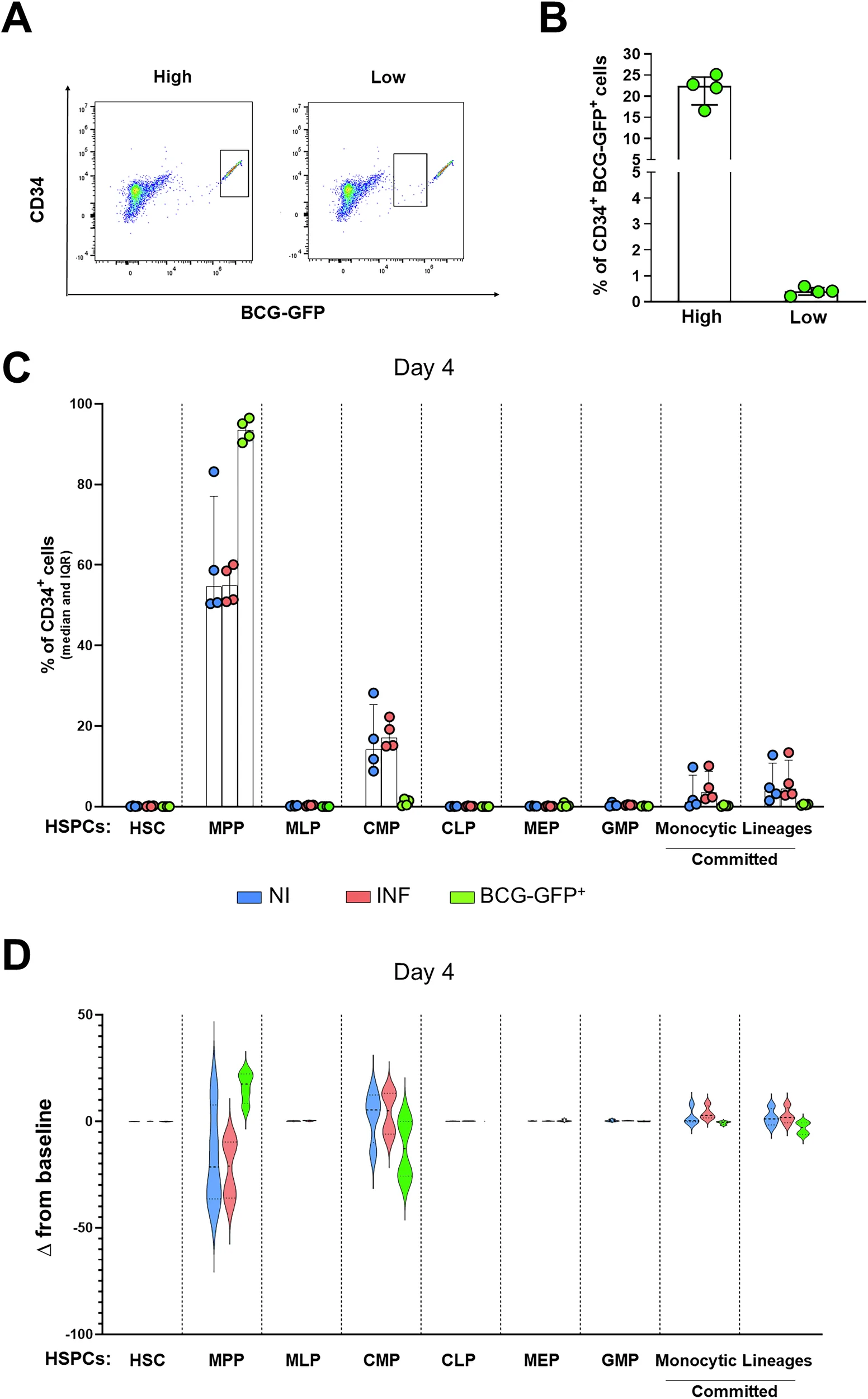
BCG infected CD34^+^ cells with a multipotent progenitor (MPP) differentiation stage. **A** Representative flow cytometry plots showing BCG-GFP^high^ and BCG‑GFP^low^ cells within the CD34^+^ population at day 4 post-infection. **B** Frequency of BCG-GFP^high^ and BCG‑GFP^low^ CD34^+^ cells at day 4 post-infection. **C** Subset-specific analysis of uninfected (NI; blue), BCG-infected (INF; red) and BCG‑GFP^+^ CD34^+^ (green) cells at day 4 post-infection. **D** Violin plots showing the changes (Δ) from baseline for NI (blue), INF (red) and BCG‑GFP^+^ (green) CD34^+^ cells, calculated as the difference between the percentage measured at day 4 post-infection and the corresponding baseline value. Data are expressed as the median with interquartile range (IQR) from four independent experiments (CD34^+^ cells were isolated from four independent pooled buffy coats, each derived from five donors). Statistical analysis was performed using GraphPad Prism Software v.8, and data were analysed using the nonparametric Mann-Whitney U test. Statistical significance was defined as: (*) *p* < 0.05, (**) *p* < 0.01. HSPC Hematopoietic Stem and Progenitor cells, HSC Hematopoietic Stem Cells, MPP Multipotent Progenitors, MLP Multipotent Lymphoid Progenitors, CMP Common Myeloid Progenitors, CLP Common Lymphoid Progenitors, MEP Megakaryocyte/ Erythroid Progenitors, GMP Granulocytes/ Macrophage Progenitors.

To investigate whether virulence factors present in *Mtb* are involved in either the infection and/or the differentiation induction of CD34^+^ HPSC, we carried out parallel infections using *Mycobacterium bovis* BCG (Bacillus Calmette-Guérin) stably expressing GFP under the control of Hsp60 promoter (BCG-GFP). BCG lacks of the RD1 genomic region, which encodes the ESX-1 secretion system and its key substrates ESAT-6 and CFP-10, major virulence determinants required for host cell modulation and full pathogenicity in *Mtb* [28, 29]. We found that BCG efficiently infects CD34^+^ cells in vitro, at the same MOI of *Mtb* infection experiments (MOI = 5) (Fig. 7A). Interestingly, almost all infected CD34^+^ cells were BCG-GFP^high^, while the BCG*-*GFP^low^ were virtually absent after 4 days of culture post-infection (Fig. 7B). The presence of the CD34^+^; BCG-GFP^high^ cells is highly reminiscent of the MPP population infected by *Mtb*. Indeed, flow cytometric analysis revealed that the majority of infected cells retained an MPP phenotype, indicating that early progenitor populations are the primary targets of BCG infection (Fig. 7C, D). This observation mirrors the tropism observed for *Mtb*, indicating that permissiveness of immature CD34^+^ progenitors to infection does not occur only with fully virulent *Mtb*. Notably, in contrast to *Mtb* infection, BCG-GFP was not found in the lineage-committed CD34^+^ cells (Fig. 7C, D), thus strongly suggesting that BCG either did not infect these populations or did not induce a detectable shift in the differentiation status of CD34^+^ HSPC.

## Discussion

The potential for *Mtb* to infect progenitor cells remains under debate. In this study, we demonstrate that *Mtb* can enter, persist, and potentially replicate within circulating human CD34^+^ HSPC in vitro.

Analysis across orthogonal planes and complete 3D rotations by high-resolution three-dimensional confocal microscopy provided definitive evidence that *Mtb* localizes intracellularly in CD34^+^ cells between the membrane and the nucleus. Notably, our findings indicate that *Mtb* remains viable and that intracellular bacilli load increase over the culture period, with the estimated number of bacilli per viable CD34^+^ cell rising from day 1 to day 4 post-infection. The approximately 0.5 log_10_ CFU increase (which accounts for a 3-4 times fold rise) at day 4 over day 1, together with the higher median number of bacilli per viable CD34^+^ cell and the increased frequency of *Mtb*-GFP^+^ CD34^+^ cells, supports ongoing intracellular persistence and suggests that CD34^+^ cells are permissive to early bacterial replication. However, since gentamicin treatment does not fully eliminate extracellular bacilli, we cannot completely rule out a contribution from residual extracellular bacteria to the observed increase in intracellular CFU.

The increased fraction of CD34^+^ *Mtb*-GFP^+^-infected cells from 3.5% to 6.8% over the three days of culture could reflect non-mutually exclusive mechanisms: (i) preferential survival or expansion of infected cells relative to uninfected counterparts, or (ii) additional infection events occurring during culture for the presence extracellular bacilli in the medium, either released by lysis of infected cells and/or resisting exposure to gentamicin. The observation at day 4 post-infection that overall CD34^+^ cell viability declined similarly in infected and non-infected cultures (with about 50% mortality), together with the increased total number of intracellular bacilli, suggests that *Mtb* entry, persistence, and possible replication in CD34^+^ cells do not promote enhanced cell death. The comparable cell viability between infected and non-infected cultures further indicates that the observed changes in the subset distribution among *Mtb*-infected CD34^+^ cells are unlikely to be driven by differential survival.

Interestingly, we show that *Mtb* GFP^+^-infected cells are most abundant within the MPP subset at day 1 post-infection, with a subsequent relative enrichment among GMP and lineage-committed CD34^+^ cells at day 4. This pattern of infection implies that *Mtb* preferentially establishes itself in MPP, the predominant cell population isolated from the buffy coat at baseline ( > 80%), and that infection is associated with accelerated differentiation along the myeloid lineages.

Such a shift is consistent with the broader concept that *Mtb*-induced inflammation can bias HSPC fate toward myelopoiesis. Interestingly, the attenuated BCG retains the ability to infect early CD34^+^ progenitors, particularly at the MPP stage, yet it does not prompt the differentiation-inducing effects observed during *Mtb* infection. These findings suggest that full virulence of *Mtb* is required to reprogram hematopoietic progenitor cells and promote their transition toward lineage-committed states. Further studies are needed to sustain this hypothesis.

The predominance of *Mtb*-GFP^+^-infected cells among MPP early after infection and the increased GFP^+^ representation among GMP and lineage-committed subsets suggest that *Mtb* may both exploit and reinforce myeloid-directed differentiation programs.

In vivo, infection of CD34^+^ progenitors by *Mtb* could contribute to the increased monocyte-to-lymphocyte ratio observed in TB and TBI subjects [30] by promoting a bias toward myelopoiesis at the expense of lymphoid output. If infected progenitors are both preferentially driven toward monocyte lineage commitment and potentially expanded or mobilized, this would result in a sustained increase in circulating monocytes. At the same time, a relative reduction in lymphoid differentiation could further amplify this imbalance. Thus, *Mtb*-induced reprogramming of early hematopoietic cells may represent a mechanistic link between pathogen persistence and systemic hematological alterations characteristic of TB.

Whether this accelerated differentiation program reflects solely the direct effects of *Mtb* -specific virulence factors on lineage-regulatory networks in MPP (i.e., via pattern recognition signaling, metabolic rewiring, or chromatin accessibility changes) [22], or also involves indirect mechanisms driven by infection-induced autocrine/paracrine cytokines in culture, remains to be determined.

*Mtb*-GFP^+^-infected cells segregated into two distinct fluorescence-intensity states. MPP largely exhibited a high-intensity GFP signal (*Mtb*-GFP^high^), whereas lineage-committed progenitors showed markedly lower fluorescence (*Mtb*-GFP^low^). These remarkable differences in fluorescence intensity between MPP and lineage-committed *Mtb*-infected cells may reflect relevant aspects of *Mtb* infection of CD34^+^ cells. Three different explanations may be considered to explain the *Mtb*-GFP^low^ pattern observed in the lineage-committed cells: (a) lower activity of the hsp60 promoter in these cells; (b) GFP-specific properties; (c) lower *Mtb* burden. The first two options are unlikely to explain the change since hsp60 is a constitutive, strong promoter in *Mtb* and we obtained similar results when we used the *Mtb*-DsRed fluorescent strain (supplementary Fig. 1). Conversely, the difference between the MPP-*Mtb*-GFP^high^ and the lineage-committed-*Mtb*-GFP^low^ patterns may be associated with a reduced bacterial burden in the latter, which may also reflect a decreased bacterial load during the transition from MPP to downstream populations. Hence, MPP may harbor more bacilli per cell, resulting in stronger GFP signals. As differentiation progresses, daughter cells might inherit fewer bacteria or eliminate part of the bacillary burden through enhanced antimicrobial mechanisms. In this scenario, we hypothesize that the lower bacilli burden in the lineage-committed CD34^+^ cells enables *Mtb* to persist intracellularly without causing or prompting cell damage or death, thereby favoring the establishment of a cellular niche that promotes *Mtb* survival.

Analysis of immune factors secreted by CD34^+^ cells revealed that *Mtb* increases IL-8 and triggers TNF-α production as early as day 1 after infection, with this effect persisting through day 4. On the other hand, chemokines such as MIP-1α, MIP-1β, and IP-10 show significant increases only at day 4 post-infection. These immune components play crucial roles in TB immunopathogenesis and contribute to cell differentiation processes within the bone marrow [31–33]. It is interesting to note that the concentrations of these mediators on day 4 post-infection showed high variability, as highlighted by the large standard deviations. We speculate that this variability may reflect the changes associated with the shift between MPP-*Mtb*-GFP^high^ and lineage-committed *Mtb*-GFP^low^ cells. However, despite the high purity of our CD34^+^ populations (94–96%), we cannot exclude a cytokines production by minor contaminating populations. Further studies are needed to elucidate the events occurring in these *Mtb*-infected cells. Understanding these mechanisms may offer relevant insights into the complex interaction between *Mtb* and CD34^+^ cells that may drive differentiation in CD34^+^ cells, and may potentially establish a niche for latency.

Since it has been previously reported that *Mtb* can infect CD34^+^ hematopoietic progenitors [18], key methodological and biological differences must be considered when comparing those findings with our results. First, earlier work relied on umbilical cord blood (UCB)-derived CD34^+^ cells [18], whereas our study examined adult circulating CD34^+^ cells, which differ markedly in intrinsic properties. UCB-CD34^+^ cells have a higher proportion of immature progenitors and show enhanced multipotent colony-forming ability, especially in inducing CFU-GM (Colony-Forming Unit-Granulocyte/Macrophage) [26, 27]. In addition, these UCB-CD34^+^ cells exhibit a reduced ability to mount an immune response compared to CD34^+^ cells derived from PBMC [26], resulting in a cellular environment that supports higher levels of infection and intracellular persistence, conditions that are advantageous for pathogen growth. These differences likely influence cell susceptibility to *Mtb*, affecting bacterial entry, intracellular stress responses, and the capacity of progenitors to support replication. Such factors may explain why CFU recovery in our cell system were approximately 1.5‑ to 2‑fold lower than in studies using UCB‑derived CD34^+^ cells. However, an additional methodological variable may also contribute to these differences. Delgobo and colleagues did not use gentamicin treatment to remove extracellular bacteria before CFU quantification, thus not ensuring that only intracellular organisms were measured. Nevertheless, despite these variations, it is consistently observed that the *Mtb*-induced myeloid differentiation of CD34^+^ HSPC takes place regardless of whether the progenitor cells come from UCB or PBMC. This underscores a conserved biological response of early hematopoietic progenitors to infection. Importantly, CD34^+^ cells isolated from adult PBMC more closely reflect the physiological context of hematopoietic progenitors encountered by *Mtb* during natural infection in humans, as these circulating progenitors represent the actual adult hematopoietic milieu that the pathogen engages in vivo. This enhances the relevance of our findings, underscoring that the observed results are not an artefact of neonatal progenitors’ biology but a common feature of adult CD34^+^ HSPC.

Notably, consistent with our in vitro findings, *Mtb* DNA has been detected in circulating CD34^+^ cells from individuals in both high TB burden settings (Ethiopia [19]) and low burden settings such as Austria [17] and Italy [21]. Although these studies detect bacterial DNA rather than viable bacilli, our current results support the possibility that *Mtb* DNA detection may reflect the presence of live *Mtb* in circulating progenitors in humans.

Interestingly, in our study, *Mtb* DNA was also identified in patients with TB-unrelated pneumonia [21], suggesting that its detection in CD34^+^ cells could complement existing approaches for TBI diagnosis, although its specificity and clinical relevance require further investigation.

One limitation of our work is the lack of mechanisms of *Mtb* entry into CD34^+^ HPSC. *Mtb* is increasingly recognized as a pathogen capable of infecting non-phagocytic cells, thereby broadening the range of host cell types that may contribute to its persistence and dissemination. Although the infection of CD34^+^ cells is clearly documented in humans [17, 19, 21], none of the established *Mtb* entry mechanisms, such as adhesin-mediated binding, toxin-induced membrane permeabilization, or enzyme-assisted barrier remodeling [34–36], have been investigated in CD34^+^ cells.

Insights from studies of mesenchymal stem cells (MSC) indicate that *Mtb* can use alternative pathways for entering into non‑phagocytic stem cell populations. MSC internalize *Mtb* via scavenger receptors MARCO and SR‑B1, restrict intracellular bacterial replication through intrinsic autophagy, and upregulate nitric oxide as part of their antimicrobial response [37]. These findings suggest that receptor‑mediated uptake may also be relevant to CD34^+^ cells. Targeted studies to define receptor usage, uptake pathways, and microenvironmental factors that enable infection of CD34^+^ progenitor populations are required and will be addressed in further studies.

Overall, our findings indicate that *Mtb* is capable of infecting CD34^+^ cells without being effectively restricted by progenitor‑related innate immunity defence. The preferential infection and higher bacterial burden in MPP, combined with their developmental plasticity, suggest that these early hematopoietic progenitors may function as a niche that supports *Mtb* persistence.

In conclusion, these results expand the spectrum of human cell types susceptible to *Mtb* infection and raise the possibility that pathogen interaction at the HSPC level contributes to early myeloid skewing and shapes the systemic immune response during TB. Together, these insights support a model in which hematopoietic progenitors represent a previously underappreciated component of the host–pathogen interface, with potential implications for host-directed therapeutic strategies.

## Materials and methods

### CD34^+^ Cell Isolation

PBMC were isolated from buffy coats obtained from healthy donors at the Transfusional Medicine and Stem Cells Unit at the San Camillo Forlanini Hospital (Rome, Italy), under ethical approval from Comitato Etico Territoriale Lazio Area 4 (approval number 123-2024). A written informed consent was obtained from all subjects.

PBMC were separated by density-gradient centrifugation (Pancoll; PAA Laboratories GmbH, Aidenbach, Germany) and resuspended in an isotonic buffer containing bovine serum albumin (BSA) and ethylenediaminetetraacetic acid (EDTA) (MACS BSA stock solution and AutoMACS rinsing solution; Miltenyi Biotec, Bergisch Gladbach, Germany). CD34^+^ HSPC were enriched using a positive immunomagnetic selection kit (Miltenyi Biotec; Bergisch Gladbach, Germany) and passed through magnetized separation columns (Miltenyi Biotec; Bergisch Gladbach, Germany). The CD34^+^ fraction was eluted, and a second step with separation columns was performed to further increase purity. Isolated CD34^+^ cells were then washed, counted by trypan blue exclusion (Merck Life Science srl, Milan, Italy), and cultured overnight in a 12-well plate at a density of 0.5 × 10^6^ cells/mL in serum‑free and cytokine-free StemSpan SFEM medium (Stem Cell Technologies; Vancouver, BC, Canada) at 37 °C with a 5% CO_2_ humidified atmosphere. The purity of the enriched CD34^+^ population was evaluated by flow cytometry. Since CD34^+^ cells represent a rare population (0.1–0.01%) among PBMC, they were isolated from buffy coats from a pool of five donors. For longitudinal experiments, up to three buffy coats were processed simultaneously to ensure adequate cell numbers. The sample size was determined based on widely accepted practices in in vitro research, where 3–6 independent biological experiments are commonly considered sufficient to ensure reproducibility and reliability of the findings.

### *Mtb*-GFP, *Mtb*-DsRed and BCG-GFP generation

The generation of *Mtb* H37Rv strain stably expressing either the green fluorescentprotein (GFP) or red fluorescent protein mCherry (DsRed) and BCG stably expressing GFP were previously described [38–40]. *Mtb*-GFP, *Mtb*-DsRed, or BCG-GFP transformants were selected on 7H11 agar (BD Bioscience, MD, USA) supplemented with Oleic Acid Albumin Dextrose Complex (OADC, BD Bioscience, CA, USA) and with 50 μg/mL of hygromycin (Sigma-Aldrich, MO, USA). Single individual antibiotic-resistant colonies were isolated and subcultured in 7H9 media (BD Bioscience, NJ, USA) supplemented with 10% OADC (BD Bioscience, NJ, USA), and 0,05% Tween 80 containing hygromycin B (50 µg/mL) and incubated at 37 °C until reaching late log phase. Aliquots (1 mL) of bacterial cultures were supplemented with 10% glycerol and stored at −80 °C until use. The infection inoculum was prepared as previously described [39].

### In vitro infection of CD34^+^ cells with *Mtb*

A total of 0.25-0.33 × 10^6^ purified CD34^+^ cells per well, at a concentration of 0.5 × 10^6^ cells/mL, were seeded in 48‑well culture plates with StemSpan SFEM medium and infected with *Mtb*-GFP, *Mtb*-DsRed or BCG-GFP at a multiplicity of infection (MOI) of 5:1 under biosafety level 3 (BSL-3) containment. Cells were then incubated for 6 h at 37 °C and 5% CO₂.

The MOI was selected based on previous studies and preliminary optimization experiments. Building on prior work by Delgobo and colleagues that used an MOI of 3 in CD34^+^ cells, and our own experiments in differentiated THP-1 cells showing no significant differences between MOI 5 and 10 (data not shown), we chose an MOI of 5 as a balanced condition to ensure sufficient infection while limiting excessive bacterial burden.

For confocal microscopy analysis, after 6 h of exposure to *Mtb*-GFP, *Mtb*-DsRed, or BCG-GFP, CD34^+^ cells were washed and processed as described below.

For flow cytometry analyses and colony-forming unit assay (CFU), after 6 h both infected and not infected CD34^+^ cells were washed and treated with gentamicin (50 µg/mL) (Merck Life Science srl, Milan, Italy) for 2 h to kill extracellular, non-phagocytosed bacteria. Subsequently, CD34^+^ cells were washed with Dulbecco’s phosphate-buffered saline (PBS) (Euroclone, Milan, Italy), resuspended in StemSpan medium alone, and incubated for 14 h (day 1 post-infection) or 96 h (day 4 post-infection).

At the indicated post-infection time points (6 h, 1 day, and 4 days), culture supernatants were collected and filtered through 0.2-µm low‑protein‑binding filters (Merck Life Science srl, Milan, Italy), by spinning at 2700 g for 2 min. Supernatants were then stored at −80 °C until further analysis.

### Confocal microscopy and immunofluorescence

After 6 h of either *Mtb*-GFP or *Mtb*-DsRed infection, CD34^+^ cells were harvested, washed extensively with PBS, resuspended in 10 µL of PBS, placed on charged glass slides and allowed to adhere by air‑drying.

Cells were then fixed with 4% paraformaldehyde (PFA) for 20 min to completely inactivate *Mtb*, blocked with 5% BSA for 20 min, washed twice in PBS. For *Mtb*-GFP analyses cells were stained for 2 h at +4 °C with an BV421-conjugated anti-human CD34 antibody (BD Biosciences, San Jose, CA, USA, #562577) diluted 1:50., washed once in PBS and nuclei were stained with DRAQ5 dye (Thermo Fisher Scientific, Waltham, MA, USA) at a dilution of 1:1000 for 30 min at +4 °C. For *Mtb*-DsRed analyses cells membranes were stained for 2 h at +4 °C with wheat germ agglutinin (WGA) Alexa Flour 488 (10 µg/mL), and nuclei with 4′,6-diamidino-2-phenylindole (DAPI) dye (1 ng/mL) both from Thermo Fisher Scientific, Waltham, MA, USA at a dilution of 1:1000 for 30 min at +4 °C. Immunostained coverslips were mounted in Prolong antifade (Thermo Fisher Scientific, Eugene, OR, USA). Images were acquired with Celldiscoverer 7 confocal microscope (Carl Zeiss Microscopy GmbH, Oberkochen, Germany) and processed using the appropriate tools of Zeiss Zen-Blue 3.6 software version (Carl Zeiss Microscopy GmbH, Jena, Germany). Lasers used were: 470 nm, 375 nm, and 640 nm. Several fields were analysed and representative results are shown.

### Colony-forming unit (CFU) assay

To quantify viable *Mtb* associated with CD34^+^ cells and determine the intracellular bacterial load, CFU assay was performed. CFU from infected CD34^+^ cells were measured in triplicate after 1 day and 4 days post-infection. Briefly, infected cell cultures were harvested, washed twice with PBS, and then lysed in 0.1 mL of PBS containing 0.1% Triton X-100 (Merck Life Science srl, Milan, Italy). Serial dilutions (from 10^−1^ to 10^−4^) were prepared in PBS supplemented with 0.05% Tween-80 (Merck Life Science srl, Milan, Italy). Fifty-microliter aliquots of each dilution were plated onto Difco^TM^ Middlebrook 7H10 agar plates (BD Biosciences, MD, USA) supplemented with 10% OADC (BD Biosciences, MD, USA) and incubated at 37˚C for 3 weeks.

After incubation, colonies were enumerated and expressed as CFU/10^6^ cells according to the following formula: CFU/10^6^ cells = Number of Colonies x (10^6^/No. of live cells plated). CFU counts were also normalized to the input cell number according to the following formula: CFU/Cell = Number of Colonies/Number of CD34^+^ *Mtb*-GFP^+^ cells.

### Flow cytometry

Purity and phenotypic analysis of CD34^+^ HSPC were evaluated by flow cytometry using a DxFLEX Flow Cytometer (Beckman Coulter, Inc., Brea, CA, USA). Briefly, cells were stained with Fixable Viability stain 700 (BD Biosciences, San Jose, CA, USA) for 10 min, washed with PBS and fixed in 4% PFA for 10 min. Cells were then washed and stained using the specific antibodies for different surface markers diluted in Brilliant Stain Buffer (BD Biosciences, San Jose, CA, USA). Phenotypic analysis of CD34^+^ HSPC was carried out by assessing surface co-expression of CD34, CD90, CD10, CD110, CD45RA, CD38, and CD14. A combined dump channel including CD3, CD16, CD56, and CD19 was applied to identify lineage-committed cells.

The following fluorescently conjugated mAbs were used: anti-CD34 Peridinin-Chlorophyll-Protein–Cyanine (PerCP-Cy5.5) (8G12 clone, #347222), anti-CD10 allophycocyanin (APC) hilite 7 (H7) APC-H7 (HI10a clone, #655404), anti-CD45RA phycoerythrin (PE)-Cy7-conjugated (L48 clone, #337186), anti-CD110 PE (1.6.1 clone, #562159), anti-CD14 Brilliant Violet™ 605 Dye (M5E2 clone, #564054), anti-CD38 BV421 (HIT2 clone, #562445), anti-CD3 BV786 (UCHT1 clone, #565491), anti-CD19 BV786 (SJ25C1 clone, #563325), anti-CD16 BV786 (3G8 clone, #563690), anti-CD56 BV786 (NCAM16.2 clone, #564058) (all from BD Biosciences, San Jose, CA, USA) and anti-CD90 APC (5E10 clone, #17090942) (eBioscience, San Diego, CA, USA). Samples were acquired using the DxFLEX Flow Cytometer, and multiple-parameter flow cytometry data were analyzed using FlowJo software (version 10.10.0, BD Life Sciences, Ashland, OR, USA) after fluorescence compensation. Frequencies of HSPC subsets were calculated as a percentage of total viable cells following hierarchical gating. The complete gating strategy is shown in supplementary Fig. 4.

### Immune factors analysis

Culture supernatants were analysed using customized MILLIPLEX® Human Cytokine/Chemokine/ Growth Factor Panel A and Panel B, according to the manufacturer’s instructions (Merck Life Science srl, Milan, Italy). The Panel A enabled the detection of the following analytes in a single well: Eotaxin, fibroblast growth factor (FGF)-2, granulocyte colony stimulating factor (G-CSF), granulocyte macrophage colony-stimulating factor (GM-CSF), interferon-gamma (IFN)-γ, IL-1α, IL-1ra, IL-2, IL-4, IL-5, IL-6, IL-7, IL-8, IL-9, IL-10, IL-12 (p70), IL-13, IL-15, IL-17A, IP-10, MCP-1, MIP-1α, MIP-1β, PDGF-bb, RANTES, TNF-α, vascular endothelial growth factor (VEGF)-A, IFN-α2 and CCL22. Panel B allowed the detection of CCL21, CXCL13, B-cell activating factor (BAFF), A proliferation-inducing ligand (APRIL), stromal cell-derived factor 1 (SDF-1), IL-21, and MIP-3β in a single well. Samples were acquired using the Bio-Plex 200 system (Bio-Rad, Hercules, CA, USA) and raw data were generated using the Bio-Plex manager software (Bio-Rad, Hercules, CA, USA).

IFN-β levels were measured using an ELLA Simple Plex Human IFN-beta assay kit (Bio-Techne, MN, USA), whereas S100A8/S100A9 Heterodimer levels were quantified using the Human S100A8/S100A9 Heterodimer Quantikine ELISA Kit (R&D systems, Bio-Techne, MN, USA).

Samples with concentrations below the detection limit of the standard curve was assigned a value of zero.

### Statistical analysis

Data were analyzed using GraphPad Prism software, version 8.0.1 (GraphPad Software, San Diego, CA, USA). Median values and interquartile ranges (IQRs) were calculated for all datasets. Statistical comparisons of unpaired data were performed using Mann–Whitney U test, whereas pairwise comparisons of paired data were conducted using the Wilcoxon Signed-Rank test. A *p*‑value < 0.05 was considered statistically significant.

Non-parametric statistical tests were used due to the inherent biological variability associated with primary cells isolated from different donors. Given the relatively small sample size and the potential for non-normal data distribution and inter-donor heterogeneity, non-parametric approaches were considered more appropriate, as they do not rely on assumptions of normality and are more robust to outliers.

## Supplementary information

Supplementary Figure 1. Assessment of CD34^+^ cell isolation purity by flow cytometry.

Supplementary Figure 2. Experiment flowchart.

Supplementary Figure 3. Intracellular localization of Mtb GFP in circulating human CD34^+^ cells.

Supplementary Figure 4. Flow cytometry gating strategy for the analysis of CD34+ cells.

Supplementary Figure 5. Increase in CD34+ Mtb-GFP+ frequency over time.

Supplementary Figure 6. CD34+ cell mortality at day 1 and 4 post-infection.

Supplementary Video 1

Supplementary Figure Legends

## Data Availability

The raw data are available in our institutional repository (rawdata.inmi.it), subject to registration. The data can be found by selecting the article of interest from a list of articles ordered by the year of publication. No charge for granting access to data is required. In the event of a malfunction of the application, the request can be sent directly by e-mail to the library (biblioteca@inmi.it).
