# Supplementary material for: Mycobacterium tuberculosis infects human peripheral hematopoietic multipotent progenitors and promotes their myeloid differentiation in vitro
Source: Cell Death Dis. 2026 Jul 31;17(1):866. doi: 10.1038/s41419-026-09122-0 (PMC13642923; doi:10.1038/s41419-026-09122-0)
Supplement: Supplementary file 1 [file 41419_2026_9122_MOESM8_ESM.docx]

**Supplementary Figure Legends**

**Supplementary figure 1. Assessment of CD34⁺ cell isolation purity by flow cytometry.** Representative flow cytometry plots showing the efficiency of CD34^+^ cell enrichment after magnetic bead isolation. Cells were first gated on forward scatter (FSC-A) and side scatter (SSC-A) to define the main cell population and exclude debris. The purity of the isolated CD34^+^ fraction was assessed based on CD34 expression (**A**) and further validated by evaluating CD14 and CD3 expression (**B**) to exclude monocyte and T-cell contamination.

**Supplementary figure 2. Experiment flowchart.** CD34⁺ cells were isolated from peripheral blood mononuclear cells (PBMC) of healthy donors using immunomagnetic beads. Purified CD34^+^ HSPC cells were infected *in vitro* with a constitutively fluorescent *Mtb* strain (H37Rv; *Mtb*-GFP; MOI=5). Six hours post-infection, cells were analyzed by confocal microscopy or treated with gentamicin for 2h and then cultured for an additional 1 or 4 days before flow cytometry, and CFU analyses. Supernatants were harvested, filtered and assessed for immune factors production. CFU: colony forming unit; GFP: Green fluorescent Protein; Mtb: *Mycobacterium tuberculosis*; HSPC: hematopoietic stem and progenitor cells.

**Supplementary Video 1. Intracellular localization of *Mtb*‑GFP in circulating human CD34⁺ cells.** A 360° rotational 3D reconstruction video of the z‑stack, illustrating the spatial relationship of the infected cells. Throughout the full rotation, the green *Mtb*‑GFP signal remains fully contained within the CD34 marker (red signal) and outside the nucleus (Blue signal), providing definitive visualization of intracellular bacterial positioning.

**Supplementary figure 3. Intracellular localization of *Mtb*‑GFP in circulating human CD34⁺ cells. (A)** Orthogonal x–z and y–z views confirming that *Mtb*‑DsRed bacilli (red signal) reside within the cytoplasmic compartment, occupying regions clearly separated from the plasma membrane (green signal) and peripheral to the nucleus (blue signal). **(B)** 2D Widefield images representing three CD34⁺ cells (red), one of which is infected by multiple *Mtb* bacilli (green). Nuclei are stained in blue.

**Supplementary figure 4. Flow cytometry gating strategy for the analysis of CD34^+^ cells.** CD34^+^ cells isolated from buffy coats of five healthy donors were infected with *Mtb*-GFP and stained with the surface markers reported in Table 1. Debris and doublets were excluded, and live cells were identified using a viability dye. Live and *Mtb*-GFP^+^ CD34^+^ cells were then subdivided into Lin^-^ and Lin^+^ populations. Lin^-^ cells were further gated based on CD38 expression and classified into HSPC subsets using CD45RA, CD90, CD10 and CD110. Lin^+^ cells were analysed based on CD45RA, CD14 and lineage marker expression. Gates were defined using appropriate negative controls. The gating strategy shown is representative of six independent experiments. HSPC: Hematopoietic Stem and Progenitor cells; HSC: Hematopoietic Stem Cells; MPP: Multipotent Progenitors; MLP: Multipotent Lymphoid Progenitors; CMP: Common Myeloid Progenitors; CLP: Common Lymphoid Progenitors; MEP: Megakaryocyte/ Erythroid Progenitors; GMP: Granulocytes/ Macrophage Progenitors; Lin: Lineage.

**Supplementary figure 5. Increase in CD34^+^ *Mtb*-GFP^+^ frequency over time.** Percentage of CD34^+^ *Mtb*-GFP^+^ cells from day 1 to day 4 post-infection. Data are presented as paired line charts from three independent experiments (CD34^+^ cells were isolated from three independent pooled buffy coats, each derived from five donors).

**Supplementary figure 6. CD34^+^ cell mortality at day 1 and 4 post-infection.** **(A)** Mortality of *Mtb*-GFP infected CD34^+^ cells (INF; red), compared to uninfected cells (NI; blue) at day 1 and day 4 post-infection. Cell mortality was assessed by flow cytometry based on viability dye staining and is shown as percentage of dead cells. Data are expressed as median with interquartile range (IQR) from six independent experiments (CD34^+^ cells were isolated from six independent pooled buffy coats, each derived from five donors). **(B)** Representative flow cytometry plots showing viability dye staining in NI and INF cells at day 1 and 4 post-infection.
